# Large-scale and high-confidence proteomic analysis of human seminal plasma

**DOI:** 10.1186/gb-2006-7-5-r40

**Published:** 2006-05-18

**Authors:** Bartosz Pilch, Matthias Mann

**Affiliations:** 1 Center for Experimental BioInformatics (CEBI), Department of Biochemistry and Molecular Biology, University of Southern Denmark, Campusvej 55, DK-5230 Odense M, Denmark; 2 Department of Proteomics and Signal Transduction, Max Planck Institute for Biochemistry, Am Klopferspitz 18, D-82152 Martinsried, Germany

## Abstract

**Background:**

The development of mass spectrometric (MS) techniques now allows the investigation of very complex protein mixtures ranging from subcellular structures to tissues. Body fluids are also popular targets of proteomic analysis because of their potential for biomarker discovery. Seminal plasma has not yet received much attention from the proteomics community but its characterization could provide a future reference for virtually all studies involving human sperm. The fluid is essential for the survival of spermatozoa and their successful journey through the female reproductive tract.

**Results:**

Here we report the high-confidence identification of 923 proteins in seminal fluid from a single individual. Fourier transform MS enabled parts per million mass accuracy, and two consecutive stages of MS fragmentation allowed confident identification of proteins even by single peptides. Analysis with GoMiner annotated two-thirds of the seminal fluid proteome and revealed a large number of extracellular proteins including many proteases. Other proteins originated from male accessory glands and have important roles in spermatozoan survival.

**Conclusion:**

This high-confidence characterization of seminal plasma content provides an inventory of proteins with potential roles in fertilization. When combined with quantitative proteomics methodologies, it should be useful for studies of fertilization, male infertility, and prostatic and testicular cancers.

## Abstract 2

The high-confidence identification of 923 proteins in seminal fluid provides an inventory of proteins with potential roles in fertilization.

## Background

Seminal fluid is the liquid component of sperm, providing a safe surrounding for spermatozoa. At pH 7.35-7.50, it has buffering properties, protecting spermatozoa from the acidic environment of the vagina. It contains a high concentration of fructose, which is a major nutriment for spermatozoa during their journey in the female reproductive track. The complex content of seminal plasma is designed to assure the successful fertilization of the oocyte by one of the spermatozoa present in the ejaculum.

Seminal plasma is a mixture of secretions from several male accessory glands, including prostate, seminal vesicles, epididymis, and Cowper's gland. The average protein concentration of human seminal plasma ranges from 35 to 55 g/l making it a rich as well as an easily accessible source for protein identification. Nevertheless, seminal plasma has the feature common to many other body fluids, that it is characterized by a high dynamic range of protein abundance, making low-abundance components difficult to analyze.

In addition to the general physiological importance of knowing the composition of seminal fluid, medical interest centers on two main areas: infertility and prostate cancer. Male infertility is a widespread medical condition with large societal and emotional costs. Since seminal fluid has important roles in spermatozoan survival and overall fertilization success, its impairment can be directly connected to infertility [1]. In-depth knowledge of the seminal proteome would thus be of great interest in this respect. After lung cancer, prostate cancer is the second leading cause of cancer death in American men [2]. Prostate-specific antigen (PSA) is a widely used biomarker for this disease, but the PSA test is relatively unspecific (see for example [3]). Potentially, seminal fluid could contain biomarkers for prostate cancer. In addition, being produced by different male accessory glands, it might be an excellent source of information about developing testis cancers. Therefore, it is important to thoroughly investigate and classify the protein content of seminal fluid.

Attempts at identifying constituents of seminal plasma have a long history. Several of its components, such as phosphatases, aminopeptidases, glycosidases, hyaluronidase, and mucin, have been known for more than 40 years [4]. Two-dimensional (2D) gel electrophoresis coupled with immunostaining was the method of choice in the pre-proteomics era to visualize the whole proteome (see for example [5]). Unfortunately, despite the large number of proteins resolved on the gels, protein spots were typically not identified in such studies. In recent years, 2D gel studies have been combined with mass spectrometric (MS) identification of protein spots changing in abundance in different clinical stages related to infertility [6,7]. The cellular component of the human ejaculum (the spermatozoa) has also been studied by 2D gel electrophoresis [8] and one study reported a change of 20 spots in infertile patients [9]. A recent study of seminal plasma, employing 2D and 1D gel electrophoresis and both matrix-assisted laser desorption/ionization time-of-flight mass spectrometry (MALDI-TOF-MS) and liquid chromatography tandem mass spectrometry (LC-MS/MS), reported the identification of 61 different proteins [10]. Another group reported analysis of prostasomes, secretory particles present in seminal plasma [11]. This study was performed with 1D gel electrophoresis followed by MS analysis. A total of 139 proteins were reported, but many of them were identified only with a single peptide and with low identification scores.

Many of the body fluid proteomics projects published recently use LC combined with ion-trap MS. Although the ion trap is very sensitive, the accuracy of mass measurement is low, which can compromise unambiguous identification of proteins [12]. To increase the certainty of identification, high mass accuracy instrumentation and thorough statistical treatment of MS data can be employed. In particular, recent advances in instrumentation included a novel linear ion trap (LTQ) with a high capacity and sequencing speed that has been coupled to a Fourier transform ion cyclotron resonance analyzer (FTMS) (LTQ-FT). This instrument combines high sensitivity and fast sequencing cycles with very high mass accuracy and resolution [13]. These features also simplify work with samples of high complexity. We have shown previously that average absolute mass accuracy using selected ion monitoring (SIM) scans is in the sub parts per million range [14]. The LTQ additionally allows routine use of two consecutive stages of MS fragmentation (MS/MS/MS or MS^3^), which further dramatically increases confidence in protein identification [15]. Importantly, the combination of very high mass accuracy and MS^3 ^makes it possible to confidently identify proteins on the basis of a single peptide.

These technological advances have not yet been applied to body fluid proteomics, and compared to human plasma the other body fluids, including seminal plasma, have received relatively little attention from the scientific community. We reasoned that a thorough analysis of body fluids in general and seminal plasma in particular may prove useful as a reference for future studies in basic physiology as well as for biomarker discovery.

Here we use state-of-the-art proteomic methods to investigate seminal plasma proteins in depth and present the most extensive analysis of human seminal plasma. We report the identification of 923 proteins in seminal plasma derived from a single person. Roughly a quarter of all proteins were identified with one peptide only, 'rescued' by MS^3 ^analysis. Around 25% of all characterized proteins are annotated as being secreted. We provide a brief overview of molecular functions of identified proteins based on gene ontology analysis. Extensive Swiss-Prot database analysis revealed that only 10% of the identified proteins were previously described as derived from the male reproductive tract. This high-confidence collection of proteins actually present in human seminal plasma can serve as a reference for future biomarker discovery.

## Results

### Measurement of the seminal plasma proteome

**Figure 1:**
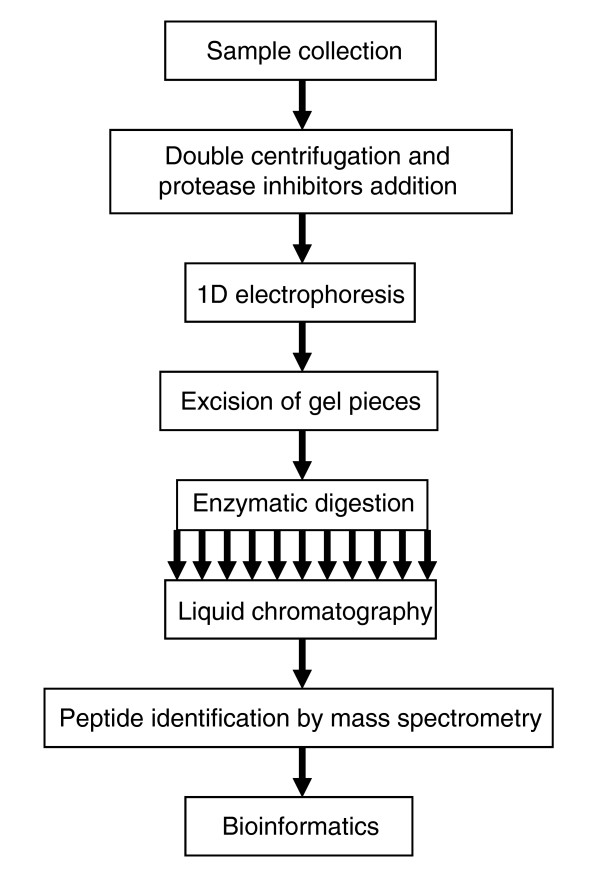
An overview of the procedure used for identification of the seminal plasma proteome.

The outline of the experimental approach is shown in Figure 1 (see Materials and methods for details). Briefly, we collected three ejaculates from a single donor. PSA is a chymotrypsin of the kallikrein subfamily and is the most potent among numerous proteases in human semen. To avoid nonspecific proteolysis occurring in semen during liquefaction, the sample was centrifuged immediately after collection and a cocktail of proteases inhibitors was added within few minutes of ejaculation. To achieve the best possible protein coverage we chose to perform straightforward 1D SDS-PAGE of seminal fluid separated from its cellular content. The lack of elaborate biochemical purification procedures ensured that there was no discrimination against certain classes of proteins in the sample before MS analysis. This is in contrast to 2D gel procedures previously applied to this body fluid, which tend to selectively loose hydrophobic, very acidic, and very basic proteins. Each of the three resulting 1D gels was excised into 14 slices covering the whole gel lane and spaced to represent roughly similar amounts of protein as judged by Coomassie staining. Gel slices were digested with trypsin to liberate peptides and these were analyzed by LC coupled to a high-performance mass spectrometer, the LTQ-FT. LC gradients lasted either 100 or 140 minutes. Altogether, 42 LC MS runs were performed and more than 50,000 MS/MS spectra were obtained (after removing unassigned spectra after database search). The mass spectrometer was programmed to perform survey scans of the whole peptide mass range, select the three most abundant peptide signals and perform SIM scans for high mass accuracy measurements. Simultaneously with the SIM scans, the linear ion trap fragmented the peptide, obtained an MS/MS spectrum and further isolated and fragmented the most abundant peak in the MS/MS mass spectrum to yield the MS^3 ^spectrum.

**Figure 2:**
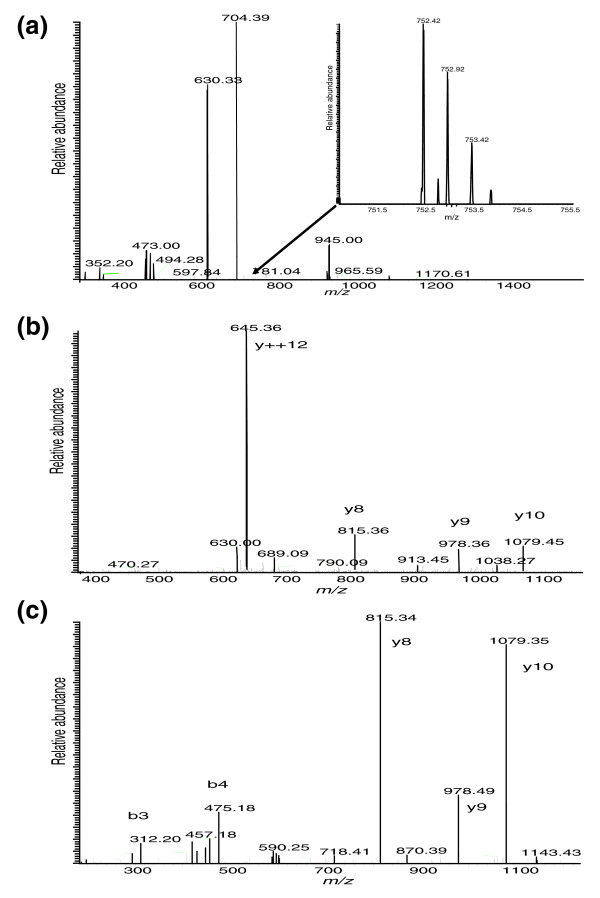
Two consecutive stages of mass spectrometric fragmentation (MS^3^). **(a) **The precursor of a peptide LTPITYPQGLAMAK (see insert) was selected for fragmentation from a full scan of mass-to-charge ratio (m/z) range. **(b) **A fragment of the above, the doubly charged y12 ion, was subsequently fragmented. **(c) **The characteristic pattern for charged directed fragmentation is observed in MS^3 ^spectra and confirms the identification of the above peptide.

Figure 2 shows an example of that procedure. The parent ion from the top spectrum is subjected to fragmentation. The relatively poor-quality MS/MS spectrum by itself would have resulted in a low identification score in a database search. The most intense fragment in the MS/MS spectrum was selected for the second round of fragmentation. The resulting spectrum, together with the MS/MS spectrum, confirms identity of the peptide and enables 'rescue' of one peptide hits as positive identifications.

### Data analysis and quality

Data from each of the LC MS runs were searched separately by a probability-based search engine (Mascot [16]). The additional information present in the MS^3 ^spectra was scored with an algorithm developed in our laboratory [15]. Both scores were added together by the open-source program MSQuant [17] (see Materials and methods), which also allowed visual inspection of the fragmentation spectra leading to peptide identifications. Data from each of the samples were combined (see Additional data file 1). Proteins were considered positively identified if they had at least two fully tryptic peptides of more than six amino acids and a Mascot score of at least 26 (95% significance level) for one of the peptides and at least 33 (99% significance level) for the other. For proteins identified by a single peptide, we required the presence of an MS^3 ^spectrum and a combined score for MS^2 ^and MS^3 ^of above 43. These criteria formally correspond to a level of false positives of *p *= 0.01 × 0.05 = 0.0005 or 5 in 10,000 if two peptides are identified and the peptides are independent. If one peptide is identified, the level of false positives is formally 1 in 1,000 for a peptide at the lowest score of 43. We also manually checked MS^2 ^and MS^3 ^spectra for all proteins identified by a single peptide. To test the level of false positives in our dataset experimentally, we performed a decoy database search (see [18] for a review). In this approach peptides are matched against the normal peptide database and against a database consisting of sequence-reversed entries. We have applied the same criteria as for the forward database search and have obtained no false-positive identifications of proteins by two peptides. From the queries with MS/MS and MS^3 ^spectra, two false-positive peptides were found, but only one passed manual inspection. We conclude that our dataset contains very few or no false-positive identifications.

Whereas trypsin is an extremely specific protease, and we therefore searched only for fully tryptic peptides [14], it was possible that kallikrein proteases, which have chymotryptic-like activity, would lead to many unassigned fragmentation spectra. However, additional database searches with fully chymotryptic specificity did not lead to additional protein hits, making it unlikely that this was the case.

To prepare a final list of proteins, we used Protein Center [19], a program to analyze the results of proteomic experiments bioinformatically. In particular, Protein Center assigns peptide identifications to proteins, resolving ambiguities resulting from peptides matching different members of protein families. Information about which protein was identified in which sample is also kept (see Additional data file 1). Protein Center also curates the identified proteome for signal peptides, transmembrane regions, and alternative splicing, and allows analysis of biological function and cellular roles. Results of Protein Center analysis, including the occurrence of proteins in one, two, or three samples and bioinformatic annotation, can be found in Additional data file 1.

### Main proteins found in the seminal fluid proteome

Although we did not use a quantitative MS format, protein abundance can be estimated very roughly by the number of peptides identifying each protein (or more accurately by the number of peptides observed divided by the number of theoretically observable peptides [20]). Among the most abundant proteins, there were no truly surprising findings. These are proteins secreted by seminal vesicles, the so called gel-forming proteins: fibronectin, semenogelin I, and semenogelin II [21]. Cleaved by kallikrein-like protease, they form a viscous gel entrapping spermatozoa immediately after ejaculation. Another highly expressed seminal vesicle protein is lactoferrin, which stays in solution and may have an antimicrobial role in seminal plasma. All three chains of heterotrimeric laminin were also highly abundant in seminal plasma. Serum albumin, the predominant element of human plasma, is also an important constituent of seminal plasma, having a role as a sink for cholesterol, which is removed from the sperm membrane during capacitation [22].

### Subcellular localization

After applying stringent criteria for protein detection, we report the identification of 923 proteins obtained by adding the results from three different samples from a single person (see Additional data file 1). For an overview of this proteome we used the GoMiner program package [23] as well as a script that retrieves data from the Swiss-Prot database for each identified protein. GoMiner provides a general view of protein localization and function whereas the Swiss-Prot database provides additional information concerning tissue expression as well as links to the literature.

According to GoMiner, 52% of catalogued proteins have been assigned a subcellular location. Of those, 78% were cellular and 25% were reported as extracellular or secreted (note that GoMiner categories are overlapping.) This is a much larger percentage than the 8% of proteins predicted to be secreted in the whole human proteome. Why are a majority of proteins not annotated as secreted? Seminal plasma contains membrane-enveloped secretory vesicles called prostasomes that are not removed by our sample preparation. They are a rich source of intracellular proteins with important roles in sperm survival and we have identified several prostasomal markers (see below). Furthermore, it is well known that body fluids contain proteins that result from epithelial shredding. For example, human plasma is thought to contain thousands of such 'leakage proteins' [24]. In the case of seminal fluid, these epithelial cells originate from the male accessory glands as well as the ductal tubes. Such proteins do not necessarily have a functional role in the body fluid, but might prove informative in the context of cancer biomarker discovery. Obviously, complete coverage of intracellular leakage proteins is unrealistic, as it would necessitate identification of essentially the whole epithelial proteome in the sample. Moreover, even though the sample was monitored under the microscope after each centrifugation and no spermatozoa were detected, we cannot rule out that some were disrupted during sample preparation.

### Molecular function

**Figure 3:**
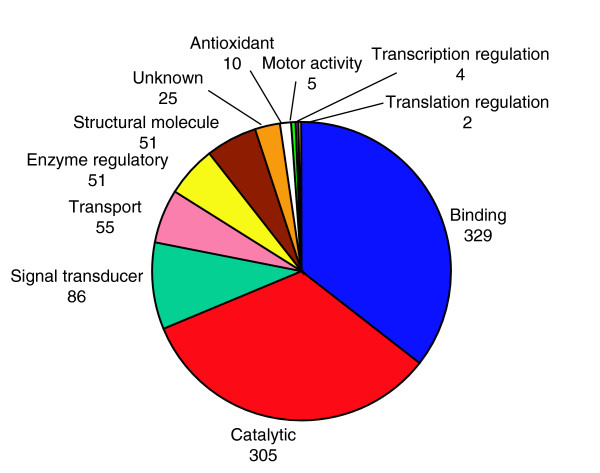
GoMiner analysis of the molecular function of identified proteins.

Figure 3 presents the GoMiner analysis for molecular function. From 595 proteins that were assigned a molecular function, 307 are engaged in catalytic activity. An additional 51 proteins are classified as their regulators, implicating 60% of the seminal fluid proteome in enzymatic activity. The number of enzymes present in seminal plasma should not surprise, given the task that seminal plasma enzymes perform. First, they need to digest a strong seminal clot formed within moments after ejaculation. The protein responsible for this is kallikrein-like protease 3 (hK3) or PSA [25]. It is likely that other proteases are involved in that process as well. Of all identified enzymes, 184 belong to the class of hydrolases, which in turn contains 75 peptidases (over 8% of all identified proteins). These digestive enzymes need to be strongly regulated to prevent unwanted proteolysis and we report identification of 35 protease inhibitors (almost 4% of all identified proteins), of which 33 are the serine-type endopeptidase inhibitors known as serpins (Table 1). One of them is a major inhibitor of PSA activity, α_1_-antichymotrypsin, a protein that complexes nearly all the PSA present in blood but whose complexes with PSA in seminal plasma are not detectable [3]. The number of proteases and protease inhibitors in seminal plasma show the importance of this system in this body fluid.

There are 86 signal transduction molecules in our proteome, forming the next largest functional group and representing more than 9% of all proteins with an annotated function. That group contains 19 Ras-related small GTPases, Rab, and Rab-related proteins, which have previously been identified in prostasomes [11]. Another subclass of enzymes is composed of seven protein kinases and nine phosphatases. The next largest groups of proteins are 55 transporter proteins and 51 structural molecules (each comprising almost 6% of the total). Even though the largest number of proteins was assigned a binding function, we believe that in many cases this function is auxiliary to a more important role of that protein which can be related to, for example transport or enzymatic activity.

### Biological processes

In GoMiner analysis of biological processes, the effect of nonexclusive assignment of proteins to different groups is most pronounced. Nevertheless, there are some interesting sets of proteins engaged in well characterized processes. The largest category is composed of 322 proteins (59% of all those given a biological function) that are involved in metabolism. This broad category contains hundreds of the above-mentioned enzymes, notably proteases, as well as enzymes involved in basic cellular processes such as glycolysis (17 proteins).

A large group of 48 proteins was assigned a role in immune responses. The seminal plasma was previously shown to suppress induction of cell-mediated cytotoxicity [26] as well as to protect spermatozoa from female humoral response. We found seven proteins involved in the regulation of these functions - members of either the classical or the alternative complement pathways. The suppression of immunity is necessary to protect spermatozoa from attack by the female immune system and to prevent immunization of the female reproductive tract against semen. A total of eight proteins are involved in blood clotting (hemostasis), such as Von Willebrand factor or tissue factor pathway inhibitor, which supports suggestions that human semen contains a functional hemostatic system [27,28].

## Discussion

### High-confidence and high-coverage analysis of the seminal fluid proteome

**Figure 4:**
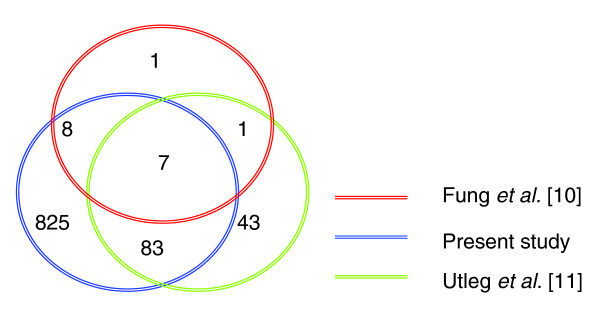
A comparison between proteins identified in the present study and two proteomics datasets published recently [10,11].

Despite physiological and medical interest in seminal plasma, previous studies trying to cover large numbers of proteins fell short of providing in-depth and high-confidence identifications of seminal proteins. Methods based on 2D gel electrophoresis revealed many protein spots, as well as quantitative changes in normal and impaired spermatogenesis [6], but only a very small number of identified proteins. More recently, low-resolution MS methods identified more proteins in seminal fluid and prostasomes [10,11]. In the present work, we used advanced MS technology and described over 900 proteins in seminal fluid, about a tenfold increase on the numbers reported previously. Peptides were identified with very high mass accuracy and with two consecutive stages of peptide fragmentation, such that the false-positive rate in our dataset is close to zero. Moreover, as proteins were solubilized and separated by 1D SDS PAGE, the dataset is not biased against hydrophobic or highly charged proteins. A comparison between our data and previously described proteomes using Protein Center is presented in Figure 4. Our dataset almost completely encompasses the proteins found by Fung *et al*. [10] and shows good overlap with Utleg *et al*. [11], given that ambiguous protein identifications were included in those data.

### Origin of proteins in the seminal fluid

Our analysis found the proteins classically known to be present in seminal fluid, including the highly abundant gel-forming proteins. Analysis of identified proteins revealed extracellular and intracellular proteins. The large proportion of proteins annotated by GoMiner to be extracellular contains many of the proteins secreted by the male accessory glands as well as extracellular matrix proteins. These are proteins required for the classical functions of seminal fluid. A second class of proteins originates from prostasomes, membrane-enclosed structures in seminal fluid that support and fuse with spermatozoa. A third class of proteins is present as a result of epithelial shredding. Epithelial cells that are abraded from the tissue surface can shed their contents into the seminal fluid. Such processes are well known from other body fluids, and in the context of the plasma proteome these proteins are thought to be potential biomarkers for disease affecting diverse tissues. In this class of leakage proteins, low amounts of any intracellular proteins from epithelial cells can potentially be present.

We identified proteins known to be characteristic for each of the organs contributing to the formation of seminal plasma: prostate, seminal vesicles, epididymis, and bulbourethral gland. The prostasomes mentioned above are secretions of the prostate gland. We have identified 90 out of the 139 prostasomal proteins recently published [11]. The very abundant serpin, protein C inhibitor (PCI), together with the above-mentioned gel-forming proteins and nitric oxide synthase are secreted by seminal vesicles [29]. Epididymal secretory protein E1, which is involved in the regulation of the lipid composition of spermatozoa, α-mannosidase, a range of antioxidant-system proteins such as γ-glutamyltranspeptidase and three isoforms of whey acidic protein (WAP) four-disulfide core domain protein indicate the epididymal content of seminal plasma [30]. The extremely abundant mucin in seminal fluid is a protein characteristic of Cowper's gland. Thus, the proteins we identify in our sample cover the secretions of all glands participating in the production of human seminal plasma, a fact that is important for the discovery of disease biomarkers.

### Problems with the characterization of identified proteins

A detailed functional study of the more than 900 proteins in seminal fluid is not feasible for a single laboratory. Even a detailed literature study of such a plethora of proteins sets a formidable challenge, a common problem in proteomic research. Instead, in common with other studies involving large numbers of genes - such as microarray studies - we used bioinformatics tools to obtain an overview of our results. We used the GoMiner program [23] to classify the seminal fluid proteome into functional classes, involvement in biological processes, and subcellular localization. There are, however, several caveats when using programs like these for classification. Functional annotations are still very sparse for the proteome overall, many of the functional categories are extremely broad (such as 'binding' or 'metabolism'), and proteins may be assigned to several categories, making the interpretation of percentages less than straightforward. Conversely, proteins can have different functions and this may not be reflected in the GoMiner classification. Some drawbacks are also associated even with very well annotated databases such as Swiss-Prot, which we used extensively in our analysis. Although not complete, the Swiss-Prot database provides information confirmed by direct assays and based on previous research, and is a more reliable source of information than a bioinformatics tool that basis its analyses only on protein sequences. Nevertheless, only about 10% of the total number of proteins was documented by Swiss-Prot as being expressed in a part of male reproductive system (16% when counting those expressed ubiquitously). There are many examples of proteins known to be a part of seminal plasma but not annotated as such. The most striking example is PSA, which was not given any subcellular localization or tissue specificity in Swiss-Prot. As all the proteins identified in this study belong to the seminal plasma proteome, at least the one predicted to be extracellular should be annotated as being part of the male reproductive system.

### Biological functions of seminal fluid as revealed by proteomics

What does this large and high-confidence set of seminal fluid proteins reveal about the function of this body fluid? The overall numbers and proportions of proteins in this proteome indicate that the predominant functions are in clot formation and liquefaction, and in metabolic support and protection for the spermatozoa. Immunological functions are also very important, judging from the number of proteins dedicated to this task. While these are 'classical' functions of seminal fluid, we have discovered an unprecedented number of proteins involved in each of these processes. These proteins are likely to have a function in fertilization and can now be studied in this context.

Seminal plasma has a higher concentration of sugar than blood plasma to provide energy for mitochondria-rich spermatozoa. Because of their morphology, spermatozoa have their cytoplasm reduced to a minimum and additional nutrient stocks are vital for their survival. The very high protein complexity of seminal fluid discovered in our study suggests a picture in which many of the vital functions of spermatozoa are provided by the surrounding fluid and the prostasomes, which may be packed with a plethora of enzymes. In addition, the process of fusion between prostasomes and spermatozoa has been described several times and involves the transfer of proteins as well as lipids necessary for the different tasks of the spermatozoa [31].

### The potential use of the proteomic data set for biomarker discovery

Identification of disease biomarkers is an overarching aim of large proteomics studies of bodily fluids. In the case of human seminal plasma, the aim would be the discovery of new biomarkers for prostate and testis cancers as well as identification of markers of male infertility. In the case of prostate cancer, a well known biomarker already exists - PSA. Although widely used, its diagnostic use is not unproblematic. Its concentration in blood is not sufficient to decisively diagnose cancer, as it can be confused with benign prostatic hyperplasia. Additional characterization of free versus total PSA is needed to distinguish between those two states [3]. Even though the concentration of PSA is six orders of magnitude higher in seminal plasma than in blood, straightforward MS analysis would encounter several problems in characterizing disease states. First, some studies have reported no correlation between tumor stage and grade and the amount of PSA in prostate tissue [32]. The attempt to establish that correlation in another body fluid (urine) was inconclusive [33]. In addition, there have been contradictory reports concerning the levels of PSA in blood and tissue in different cancers [3]. Clearly, quantitative MS techniques (reviewed in [34]) will be needed to establish if PSA or any of the other identified components in seminal fluid can serve as biomarkers. Although not done here, proteomics can potentially be used to distinguish PSA isoforms that may be of use in differential diagnosis [35,36]. Besides PSA, homologous human kallikrein 2, identified as an abundant protein in this study, was previously shown to be associated with prostate diseases [37]. Glutamate carboxypeptidase II (prostate-specific membrane antigen), identified with 26 peptides, and prostate stem-cell antigen are other strong indicators of prostate cancer. PCI expression is also associated with prostate cancer [38]. It should be kept in mind that biomarkers could also be discovered in seminal fluid but in clinical practice be assayed in a blood test.

The present set of seminal fluid proteins may also be an excellent resource for studies into the complex problem of male infertility. These proteins could be investigated with a view to their involvement in the reduced viability of sperm. On the other hand, if other large-scale studies implicate groups of proteins in infertility, these proteins could be checked for overlap with the proteins found here.

## Conclusion

The in-depth analysis of seminal fluid revealed over 900 proteins. These proteins provide interesting hints of the complexity and of the main functions of this body fluid. Complete functional characterization of the roles of so many proteins in fertilization surpasses the scope of any single group. Instead, we plan the creation of a publicly accessible database, which would include the data from the seminal fluid proteome, together with the results from other body fluids, initially tear fluid, the urinary proteome, and cerebrospinal fluid (see below Additional data file). The data on which this paper is based, including accurate information concerning identified peptides, is available as Additional data file 1. This data will also be part of a database that could serve as a reference for future studies. Further developments in quantitative proteomics potentially open a large field of possible investigations, especially for biomarker discovery.

## Materials and methods

### Sample collection and SDS-PAGE

Fresh ejaculate was collected from a healthy, 27-year-old Caucasian male and immediately spun down at 13,000 *g *for 5 minutes at 4°C to separate seminal fluid from spermatozoa. Phenylmethylsulphonylfluoride (PMSF, 0.2 mM), benzamidine (0.1 mM), and 1 μg/ml each of aprotinin, leupeptin, and pepstatin (Sigma, St. Louis, USA) were added to the sample to avoid digestion by powerful proteases present in seminal fluid. To ensure complete separation of cell debris or occasional spermatozoa from seminal plasma, the sample was centrifuged at 100,000 *g *for 30 minutes at 4°C. Protein concentration was assessed by Coomassie Plus assay (Pierce, Rockford, USA) and 1 mg protein was resolved on 10% NuPAGE Novex Bis-Tris gel (Invitrogen, Carlsbad, USA). The gel was cut into 14 pieces and subjected to standard in-gel trypsin digestion protocol [39]. Briefly, the pieces were washed twice with 25 mM ammonium bicarbonate/50% ethanol, dehydrated with absolute ethanol, reduced for 1 hour at 56°C with 10 mM dithiothreitol (DTT), alkylated for 45 minutes in the dark with 55 mM iodoacetamide. After extensive washing with ammonium bicarbonate and dehydratation, the 12.5 ng/μl trypsin solution (modified sequencing grade; Promega, Madison, USA) was added and the enzyme was allowed to function overnight at 37°C. The peptides were extracted with 30% acetonitrile, 3% trifluoroacetic acid (TFA) and the organic solvent was evaporated in a vacuum centrifuge. TFA was added to the final concentration of 2% and stop-and-go extraction tip purification was performed as previously described [40].

### LC-MS/MS and data analysis

The nano-high-pressure LC-MS^3 ^analysis was performed on an Agilent 1100 nanoflow system connected to a LTQ-FT mass spectrometer (Thermo Electron, Bremen, Germany) equipped with a nanoelectrospray source (Proxeon Biosystems, Odense, Denmark). The mass spectrometer was operated in data-dependent mode to automatically switch between MS, MS^2 ^and MS^3 ^acquisition. Survey spectra in the mass-to-charge ratio (m/z) range 300-1,575 were acquired in the Fourier transform ion cyclotron resonance (FT-ICR) and three most intense ions in the m/z range 450-1400 were sequentially chosen for accurate mass measurement by FT-ICR SIM. They were simultaneously fragmented in the ion trap to obtain MS^2 ^spectra. The most intense ion in the MS^2 ^spectra was selected for another round of collision-induced dissociation to obtain MS^3 ^spectra. The other MS conditions were as described previously [15].

The acquired data was searched against the International Protein Index human protein sequence database (version 3.04) with the automated database-searching program Mascot (Matrix Science, London, UK). Spectra were searched with a mass tolerance of 5 ppm for MS data and 0.5 Da for MS/MS data. Up to three missed trypsin cleavages were allowed. Carbamidomethyl cysteine was set as a fixed modification, and oxidized methionine, protein *N*-acetylation and deamidation were set as variable modifications. MS^3 ^spectra were automatically scored with MSQuant, open-source software developed in our lab [17]. This program is a validation tool parsing Mascot peptide identifications and enabling their manual and automated validation.

To prepare our protein list, our peptide identifications were subjected to very stringent filtering. Only peptides of seven amino acids or longer were accepted for identification. All of them were required to score above 26, the score calculated by Mascot to be statistically significant. For two-peptide hits, one of the peptides had to score above 33 (99% probability of being correct). In the case of one-peptide hits, MS^3 ^spectra were required and a score above 43 (99.9% probability) was required. All these peptides were manually checked as well. All the steps of the above procedure were repeated three separate times and the results were merged before the final protein evaluation. The merging of data was performed with Protein Center [19] (Proxeon), which collapses entries with at least 98% sequence homology and groups homologous sequences. Swiss-Prot data was extracted from a database by in-house software (courtesy of Gary Schoenhals).

## Additional data files

Additional data on the proteins and peptides identified in this study are available (Additional data file 1). All data are freely available at the proteome database of the Department of Proteomics and Cell Signaling of the Max-Planck-Institut for Biochemistry [41].

## Supplementary Material

Additional data file 1 The additional data consist of two worksheets containing respectively proteins and identifying them peptides. Worksheet 1 contains proteins consist of columns A to I displaying, respectively: IPI number; Accession ID (Swiss-Prot number prevailing, where it was lacking, we chose the next one from the original spreadsheet); MW - molecular mass; number of peptides with which the protein was identified; protein name; and information extracted from Protein Center software (columns F to J, respectively): gene name; number of transmembrane regions; signal peptide; alternative splicing; occurrence in three consecutive analyses. Worksheet 2 contains information about all the peptides identifying the proteins, in columns A to L, respectively: IPI number; Swiss-Prot (or other identifier); protein name; MW - molecular mass of the protein; peptide sequence; gi number; Mascot peptide score; combined Mascot peptide score and MS^3 ^score; MS^3 ^precursor; peptide length; delta mass (ppm); and charge of the peptide. Click here for file

## Figures and Tables

**Table 1:** A list of identified inhibitors of serine proteases

Accession number	Protein name	Molecular mass (Da)	Number of peptides
Q5SYA8	CD109	162,530	28
P01009	Alpha-1-antitrypsin	48,436	26
P05154	Plasma serine protease inhibitor	45,787	19
P29622	Kallistatin	50,808	15
P05155	Plasma protease C1 inhibitor	57,067	13
Q99574	Neuroserpin	46,397	13
P35237	Placental thrombin inhibitor	42,904	13
P03973	Antileukoproteinase 1	15,228	12
O43278	Kunitz-type protease inhibitor 1	58,616	10
Q8TF48	Inter-alpha (Globulin) inhibitor H5	106,524	9
O43692	25 kDa trypsin inhibitor	59,557	9
P30086	Phosphatidylethanolamine-binding protein	25,361	8
P01019	Angiotensinogen	54,465	7
Q06481-1	Amyloid-like protein 2	87,927	6
P01011	Alpha-1-antichymotrypsin	50,767	6
P05067-1	Amyloid beta A4 protein	87,914	5
P08697	Alpha-2-antiplasmin	56,917	5
P23352	Anosmin 1	77,444	4
Q14508-1	WAP four-disulfide core domain protein 2	13,953	4
P20155	Serine protease inhibitor Kazal-type 2	9,627	4
P29508	Squamous cell carcinoma antigen 1	44,594	3
P49223	Kunitz-type protease inhibitor 3	7,744	3
P01008	Antithrombin-III	53,114	2
P36955	Pigment epithelium-derived factor	48,809	2
P08185	Corticosteroid-binding globulin	45,283	2
Q8IUA0	WAP four-disulfide core domain protein 8	29,489	2
P05543	Thyroxine-binding globulin	46,637	1
P36952	Maspin	42,568	1
P02760	AMBP protein	39,886	1
P48307	Tissue factor pathway inhibitor 2	29,567	1
Q9BQN3	PREDICTED: similar to dJ601O1.1 (novel protein with Kunitz)	15,847	1
